# A huge retropharyngeal lipoma: a rare cause of dysphagia: a case report and literature review

**DOI:** 10.11604/pamj.2019.33.12.18541

**Published:** 2019-05-07

**Authors:** Monia Ghammam, Jihene Houas, Mouna Bellakhdher, Mohamed Abdelkefi

**Affiliations:** 1 ENT Department and Cervical Surgery Farhat Hached Hospital, Medicine University, Sousse, Tunisia

**Keywords:** Lipoma, retropharyngeal, dysphagia, surgery

## Abstract

Dysphagia is commonly seen after a cerebral vascular accident. It is rarely caused by lipomas of the retropharyngeal region which are rare benign mesenchymal neoplasms. We report a case of a 53-year-old man who presented with a history of ptyalism and dysphagia occurring after a brain stroke. Flexible nasal endoscopy revealed a pooling of saliva in both pyriform sinuses. Cervical and neurological examinations were unremarkable. Computed tomography (CT) scan suggested the diagnosis of retropharyngeal lipoma. The mass was resected by trans-cervical approach. The histological examination confirmed the diagnosis of a retropharyngeal lipoma. The postoperative course was unremarkable. Although lipomas in the retropharyngeal space are rare, clinicians should evoke this diagnosis when treating a patient presenting with dysphagia, even if there is a medical history of cerebral vascular accident.

## Introduction

Dysphagia is a sensation of difficulty in transferring the bolus from mouth through the esophagus into the stomach. There are a wide range of underlying causes such as neurologic diseases like stroke, a Zenker's diverticula, esophageal dysmotility, inflammation and structural abnormalities such as malignancy or external compression [1, 2]. Retropharyngeal tumors are rarely reported as causes of dysphagia; retropharyngeal lipomas being one of the rarest. These tumors grow to a large size because of the distensibility of the retro/parapharyngeal space before causing symptoms of upper aerodigestive tract obstruction [3]. In the following paper we report a case of retropharyngeal lipoma presenting with ptyalism and dysphagia in a patient with medical history of a cerebral vascular accident which was managed surgically with complete amelioration of symptoms.

## Patient and observation

**Figure 1:**
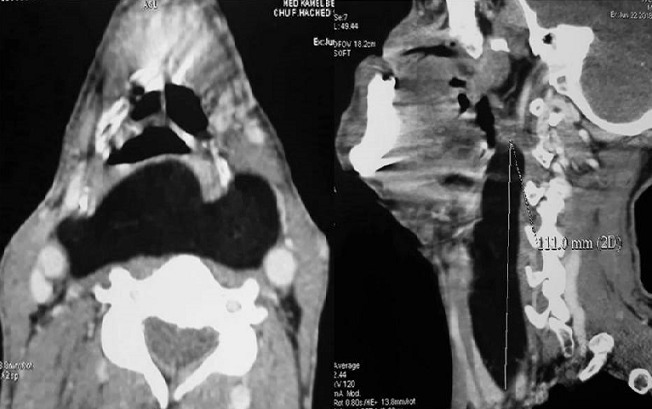
CT (computed tomography) image of the head and neck shows a homogeneous, low density mass measuring 110 cm × 73 cm × 26 cm in the posterior pharyngeal wall, extending from C2 to C7

**Figure 2:**
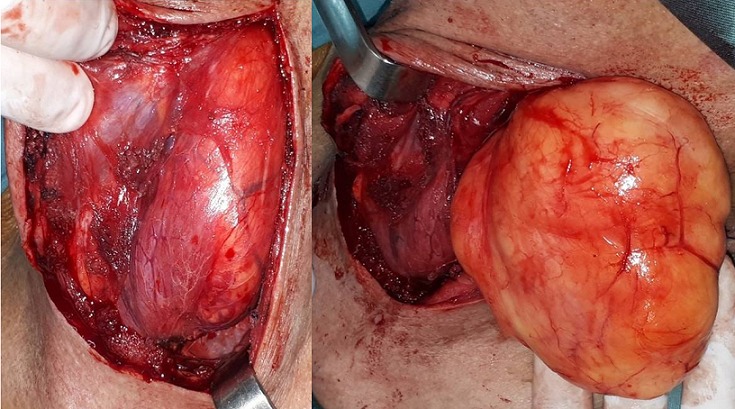
intraoperative photo shows the dissection of the mass of the retropharyngeal space

**Figure 3:**
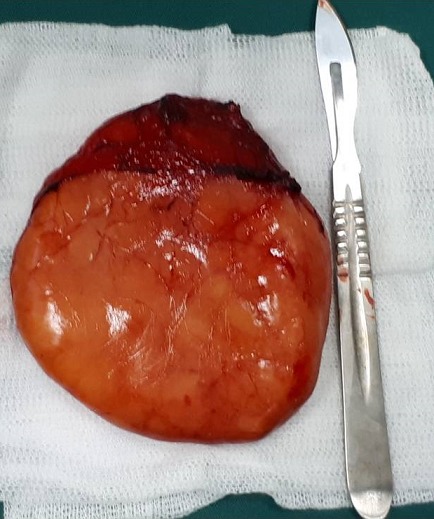
gross specimen

A 53-year-old man presented to our department with a 7-year-history of ptyalism. He also complained of progressive dysphagia for solids and liquids with intermittent choking episodes for 8 months. There were no other complaints, especially dyspnea, odynophagia, voice changes, weight loss or neurological symptoms (nasal regurgitation, fatigability or diurnal variation of dysphagia). The patient has a medical history of hypertension, an acute coronary syndrome with persistent ST-segment elevation 12 years ago and a cerebral vascular accident 7 years ago with mild left sequelae hemiparesis. On examination, there was no cervical swelling. Flexible nasal endoscopy revealed a pooling of saliva in both pyriform sinuses. Oropharynx and endolarynx exams were unremarkable. On neurologic examination, bedside screening cognitive functions, speech, cranial nerve functions, palatal movements and gag reflex were normal. Routine biochemical and hematological examinations were unremarkable. Despite patient’s history and the fact that symptoms started immediately after the stroke, an organic cause of dysphagia was suspected and a cervical CT was indicated (because of the characteristics of the dysphagia). The CT revealed a low density, homogeneous lesion measuring 7,3 cm × 2,6 cm x 11,1 cm in the posterior pharyngeal wall extending from level C2 to level C7. The mass was well defined with a few thin regular septae and did not enhance after injection of contrast medium. The mass was displacing the airway and the left thyroid lobe anteriorly and displacing the carotids laterally. There was no invasion to the neighboring muscular structures Figure 1. These findings suggested the diagnosis of a benign retropharyngeal lipoma. The intraoral approach was impossible due to the huge size of the lipoma. The patient underwent cervical excision of the lipoma under general anesthesia. The neck was incised vertically to the left of the midline and the lipoma was exposed. The tumor mass was released from surrounding tissue, such as the carotid artery, bilateral jugular vein, esophagus, left thyroid lobe and trachea. The lipoma was removed intact Figure 2. Histopathology found a mass of a 124 grams and confirmed the diagnosis of a benign lipoma without evidence of malignant change Figure 3. The postoperative course was unremarkable. No dysphagia or recurrence was observed at the last follow-up.

## Discussion

Dysphagia is common in patients with neurological disorders. In this case, the dysphagia seems to be caused by lesions in the central or peripheral nervous system or disorders of muscle or the neuromuscular junction [1]. A careful medical history and physical examination are necessary to establish the cause of the swallowing difficulty and exclude coincidental dysphagia due to organic causes [2]. Retropharyngeal lipoma is one of the exceptional organic etiologies of dysphagia. Lipomas are the most common mature adipocyte soft tissue tumors [3]. They usually occur as subcutaneous lesions with a distribution of 13% in the head and neck region. Retropharyngeal lipomas are rare tumors, with less than 50 cases reported in the literature. They may grow to considerable sizes before becoming symptomatic. They can extend from the base of the skull to the superior mediastinum [3-5]. The symptoms are predominantly depending on the affected aero-digestive tract. They include neck mass, dyspnea, snoring and sleep apnea [3-6]. The CT and Magnetic resonance (MR) imaging are helpful in the preoperative assessment of the patient [7, 8]. On CT, lipomas appear as homogeneous hypo attenuated masses. They have a CT number ranging from -50 to -150 Hounsfield values. Typically, they don't show contrast enhancement and may contain thin septae. The CT cannot definitively distinguish a lipoma from a liposarcoma. Although the incidence of malignant transformation is low, rapid changes in symptomatology or radiological findings should be alarming [7]. The MR imaging provides better tumor delineation because it has superior soft-tissue contrast resolution. On MR images, fat has typical signal intensity. On T1-weighted images, they tend to have high signal intensity that decreases with progressive T2 weighting. Fat is also suppressed with the use of fat-suppression pulses [8]. Complete surgical removal is the preferable treatment. The surgical approach changes depending on the location of lipoma [3, 4, 9]. Retropharyngeal lipoma can be managed through transoral or external approach. Transoral robotic surgery has recently been used for the excision in some cases of retropharyngeal lipoma [10]. Transoral excision is the most preferred way for surgery because of the lower postoperative morbidity, the shorter hospitalization time and the absence of scar tissue [4-11]. On the other hand, transcervical approach is an option for large lipomas because it gives an easier access to the mass, nevertheless post-surgery morbidity is greater than transoral approach and there can be a risk of damaging cranial nerves and carotid artery [3, 11, 12]. After surgical excision, histopathological confirmation is essential to distinguish between lipoma and liposarcoma. Lipomas are composed of lobulated, slow-growing, mature adipose tissue, having a minimal connective tissue stroma. They are commonly enclosed in a thin, fibrous capsule.

## Conclusion

Dysphagia seems to be a common complaint after stroke. The challenge is to distinguish between a mild post stroke dysphagia and other organic causes of dysphagia. Retropharyngeal lipomas are rarely reported as an etiology. They are usually asymptomatic. The main symptoms are dysphagia and dyspnea. The radiological features of a lipoma can be ascertained using either CT or MR imaging. The definitive treatment is surgical excision by transoral or external approach.

## Competing interests

The authors declare no competing interests.
